# Altered networks in bothersome tinnitus: a functional connectivity study

**DOI:** 10.1186/1471-2202-13-3

**Published:** 2012-01-04

**Authors:** Harold Burton, Andre Wineland, Mousumi Bhattacharya, Joyce Nicklaus, Keith S Garcia, Jay F Piccirillo

**Affiliations:** 1 Department of Anatomy & Neurobiology and Department of Radiology, Washington University School of Medicine, St. Louis, Missouri 63110, USA; 2 Department of Otolaryngology - Head & Neck Surgery, Washington University School of Medicine, St. Louis, Missouri 63110, USA; 3 Department of Anatomy & Neurobiology, Washington University School of Medicine, St. Louis, Missouri 63110, USA; 4 Department of Otolaryngology - Head & Neck Surgery, Washington University School of Medicine, St. Louis, Missouri 63110, USA; 5 Department of Psychiatry, University of Texas-Southwestern, Austin, Texas, 75390, USA; 6 Department of Otolaryngology - Head & Neck Surgery, Washington University School of Medicine, St. Louis, Missouri 63110, USA

**Keywords:** tinnitus, human, MRI, connectivity

## Abstract

**Background:**

The objective was to examine functional connectivity linked to the auditory system in patients with bothersome tinnitus. Activity was low frequency (< 0.1 Hz), spontaneous blood oxygenation level-dependent (BOLD) responses at rest. The question was whether the experience of chronic bothersome tinnitus induced changes in synaptic efficacy between co-activated components. Functional connectivity for seed regions in auditory, visual, attention, and control networks was computed across all 2 mm^3 ^brain volumes in 17 patients with moderate-severe bothersome tinnitus (*Tinnitus Handicap Index: average *53.5 ± 3.6 (range 38-76)) and 17 age-matched controls.

**Results:**

In bothersome tinnitus, negative correlations reciprocally characterized functional connectivity between auditory and occipital/visual cortex. Negative correlations indicate that when BOLD response magnitudes increased in auditory or visual cortex they decreased in the linked visual or auditory cortex, suggesting reciprocally phase reversed activity between functionally connected locations in tinnitus. Both groups showed similar connectivity with positive correlations within the auditory network. Connectivity for primary visual cortex in tinnitus included extensive negative correlations in the ventral attention temporoparietal junction and in the inferior frontal gyrus and rostral insula - executive control network components. Rostral insula and inferior frontal gyrus connectivity in tinnitus also showed greater negative correlations in occipital cortex.

**Conclusions:**

These results imply that in bothersome tinnitus there is dissociation between activity in auditory cortex and visual, attention and control networks. The reciprocal negative correlations in connectivity between these networks might be maladaptive or reflect adaptations to reduce phantom noise salience and conflict with attention to non-auditory tasks.

## Background

The objective of the current study was to examine cortical functional connectivity associated with auditory cortex in patients with moderate-severe tinnitus. Networks associated with the auditory cortex were examined because idiopathic, non-pulsatile subjective tinnitus involves hearing noise in the absence of acoustic stimulation [1,2] and particularly affects central auditory processing [3-5]. Damage to primary auditory inputs often precedes tinnitus and possibly precipitates changes in the firing patterns of neurons in the auditory system [4,6-9] comparable to abnormal activity and altered sensory maps noted following deafferentation [10,11]. A better understanding of functional connectivity in tinnitus is important because millions experience chronic tinnitus [1] and the persistent noise disturbs the qualities of life in ~20% with bothersome tinnitus [7,12,13]. A hypothesized contributory mechanism is that chronic tinnitus might alter synaptic efficacy [14] leading to reorganization in co-activity between cortical auditory and other sensory networks. Additionally, dealing with the cognitive distractions caused by phantom noises might conflict with networks that enable focusing of attention and prevent involuntary switching to salient yet phantom noises. Thus, tinnitus might deplete cognitive resources [15] and compromise attending to visual tasks such as reading. A consequence of a dual-task situation involving thoughts related to tinnitus and performing other demanding tasks might alter functional connectivity between cortex for auditory, visual and attention processes. Furthermore, tinnitus might affect the default mode network (DMN), which is especially active at rest [16-19] and shows reduced activity during sensory tasks [20,21]. Tinnitus as a condition involving sensations of persistent phantom auditory might then act similarly in reducing DMN activity.

Prior behavioral evidence indicates that tinnitus disrupts the allocation of attention to non-auditory stimuli [22]. Thus, phantom noises, like chronic pain, involuntarily compete for attention resources [15,22,23]. Demonstrations of a role for attention in tinnitus include reductions in experienced tinnitus by training to habituate tinnitus salience and to focus on other sensations [24]. Cognitive distraction also diminishes tinnitus and lowers auditory cortex activity [25]. There is additional evidence that tinnitus sufficiently conflicts with non-auditory sensory processes to alter concentration and focus [26-28], thereby lowering accuracy on attention demanding tasks [15,22,29,30].

Neural evidence supporting the notion of changes in non-auditory cortex was transient diminution of tinnitus in some patients following global reductions in cortical activity after intravenous lidocaine. In the affected cases, positron emission tomography showed lower regional cerebral blood flow (rCBF) in the left middle and inferior temporal cortex [31] and right middle frontal gyrus (rMFG), parietal cortex, and right temporal-parietal junction (rTPJ) [32]. The latter three regions are of interest because they include areas involved in attention. The dorsal parietal intraparietal sulcus in parietal cortex serves voluntary focusing of attention; the ventral rTPJ responds when attention involuntarily shifts to a salient, unexpected stimulus; and the posterior aspect of rMFG links activity in dorsal and ventral attention networks [33,34]. Thus, finding that lidocaine induced rCBF reductions in these regions support the hypothesis that some tinnitus abnormalities might involve components of the attention network.

Previously, interregional temporal correlations of resting state, low frequency (< 0.1 Hz) blood oxygenation level-dependent (BOLD) activity have revealed widely distributed, coherent activity in normal individuals [16,35,36] and those with neurological pathologies [37-40]. Utilizing comparable imaging and analysis techniques in tinnitus and age-matched individuals provided a means of assessing potential differences in cortical networks in these two groups.

Temporal correlations between a seed region of interest and the rest of the brain can show positive or negative correlations when analyzed in reference to a global brain signal [41]. Such correlated intrinsic activity can indicate directly or indirectly connected distant regions [42]. Consequently, possible differences in functional connectivity between tinnitus and controls might include changes between widely separated cortical areas without direct structural connections. In the present study, we found negative correlations reciprocally characterized functional connectivity between auditory and occipital/visual cortex in the tinnitus group. Negative correlations indicate that when BOLD response magnitudes increased in auditory or visual cortex they decreased in the linked visual or auditory cortex, suggesting reciprocally phase reversed activity between functionally connected locations in tinnitus. The results implied that in tinnitus activity suppression might reflect adaptations to reduce phantom noise salience and aid directing attention to non-auditory events.

## Methods

### Participants

Seventeen patients (mean age 53.5 SEM ± 3.6 years, 5 female), who participated in a randomized clinical trial of repetitive transcranial magnitude stimulation (rTMS) for tinnitus (ClinicalTrials.gov Identifier: NCT00567892), had tinnitus for an average of 8.3 years (± 1.9 years, range 0.5-30 years), with an average loudness of 7.6 ± 0.3 (on a 1-10 scale), and an average severity of 53.5 ± 3.6 (range 38-76) based on the Tinnitus Handicap Index (THI) [43]. Based on the THI score and previously proposed guidelines [44], 10 patients had Moderate (38-56) and 7 Severe (58-76) tinnitus. Tinnitus was bilateral in 11 and unilateral in 5 patients (4 on the right and 1 on the left). Hearing loss was minimal for 1-3 kHz tones and > 40 dB for 8 kHz in 12/17 patients (Table 1). No patients had hyperacusis. Post hoc t-tests of binaural pure tone average thresholds (PTA) found no significant differences between ears (left ear: mean 17.5, SEM ± 1.9 dB, range 0-65dB; right ear: mean 35.5 ± 3.1 dB, range 0-75 dB). No included patients had a Beck Depression Inventory-II > 18 [45] or other psychiatric or neurologic disorders. Seventeen individuals without tinnitus were age-matched to the tinnitus patients (mean age 50.6 ± 2.5 years, 10 female) and had pure tone average thresholds of ≤ 25 dB HL. All participants provided informed consent in compliance with the Code of Ethics of the World Medical Association (Declaration of Helsinki) and guidelines approved by the Human Studies Committee of Washington University.

**Table 1:** Tinnitus participant demographics

							1 kHz		2 kHz		3 kHz		8 kHz	
Tinnitus	Age	Sex	Ear	THI	Loud	yrs	R	L	R	L	R	L	R	L
1	54	F	B	70	7	2	5	5	0	10	10	10	60	65
2	48	M	B	76	8	1	5	5	5	0	0	5	15	15
3	47	M	B	38	8	8	10	5	5	0	25	10	55	65
6	53	M	R	68	5	3	25	15	30	20	60	45	80	55
7	59	M	B	40	7	1	22	18	20	20	25	25	45	50
10	48	M	R	40	7	2.5	10	10	5	0	35	25	55	40
11	57	M	L	72	7	10	20	25	20	20	15	30	40	40
15	53	M	B	40	8	5	30	25	60	45	75	65	50	35
16	58	M	L	70	9	0.5	10	5	10	15	45	30	65	75
17	59	M	B	72	8	9	0	5	15	20	45	55	85	85
19	52	F	B	40	9	2.5	15	10	10	10	20	35	30	35
20	52	F	R	52	9	9	70	20	70	15	75	15	105	40
21	42	M	B	40	7	10	0	0	0	0	10	10	10	10
24	59	M	B	52	6	30	25	30	70	65	55	65	45	50
27	58	M	R	42	8	20	15	5	55	15	75	55	50	65
37	58	F	B	60	8	11	20	15	25	25	25	40	70	85
38	52	F	B	38	8	16	5	10	5	5	5	0	15	15

Mean	53.5	5 F/12 M		53.5	7.6	8.3	17.6	12.7	22.9	17.1	35.4	30.4	52.9	47.1
SEM	1.2			3.6	.3	1.9	4.3	2.3	6.1	4.5	5.9	4.9	6.3	5.1

Participants with tinnitus had been in a study that evaluated the treatment efficacy of rTMS to the left temporoparietal junction [46]. We obtained baseline resting state imaging data for the current study prior to any rTMS treatments.

### Image Acquisition

Acquisition of magnetic resonance images was with a Siemens 3 Tesla TRIO scanner (Erlangen, Germany) and standard 12 element RF head coil. MRI headphones and ear plugs dampened sequence noises. A gradient recalled echo-planar sequence (EPI) captured images of blood oxygenation level-dependent (BOLD) contrast responses (Repetition time [TR] = 2200 ms, echo time [TE] = 27 ms, flip angle = 90°, 4 × 4 × 4 mm voxels). EPI images of the whole brain involved volume acquisitions across 36 odd-even, contiguously interleaved, axial slices aligned to the anterior and posterior commissures. Structural images included a T1-weighted magnetization prepared rapid gradient echo (MP-RAGE) sequence acquired across 176 sagittal slices (TR = 2100 ms; TE = 3.93 ms; flip angle = 7°; inversion time [TI] = 1000 ms; 1 × 1 × 1.25 mm voxels). An additional T2-weighted structural image across 36 axial slices (TR = 8430 ms, TE = 98 ms, 1.33 × 1.33 × 3 mm voxels)was in-register with the EPI and aided alignment between axial EPI and sagittal MP-RAGE image slices [47].

Three 164 frame EPI runs recorded spontaneous brain activity while participants were awake, performed no task, and kept their eyes closed in a darkened room. We spoke to participants during ~ 2 minutes between EPI runs to make certain they remained awake.

### Image Processing

EPI image corrections involved processing to compensate for systematic time and intensity slice-dependent differences from interleaved odd-even slice acquisition, to realign slices into atlas space, to band-pass filter for low frequencies, and to remove nuisance variables. Processing started with aligning the time for each slice to the beginning of each volume acquisition using sinc interpolation. Next, corrections for intensity differences between slices utilized a whole brain mean signal intensity normalized to mode 1000 across EPI runs. These time and intensity adjusted slices were realigned within and across runs using rigid body correction for inter-frame head motion [36,48-50]. The across-run-realigned slices were resampled to 2 mm^3 ^volumes (voxels) and registered to an atlas template by computing 12 parameter affine transforms between an average from the first frames of each EPI run and the atlas template [47]. An atlas template, created using MP-RAGE structural images from 12 normal middle-age individuals (mean 48 yrs., SD ± 10.7), conformed to Talairach atlas space [51] based on spatial normalization methods [52].

Atlas registered EPI images were spatially smoothed with a 6 mm full width at half-maximum Gaussian kernel and temporally band-pass filtered to remove frequencies > 0.1 Hz. BOLD signal modifications per voxel removed, through linear regression, 9 sources of nuisance variance and their associated temporal derivatives. These variables included previously computed six linear corrections for head movement, a global whole-brain signal averaged over all voxels in a fixed region of atlas space, and signals in the ventricles and white matter. Ventricles and white matter were identified across successive structural image slices in each participant using Analyze (Mayo Research Foundation, Rochester, MN). Individualized identification of ventricle and white matter structure improved isolation of signals in these regions and improved regression of spurious influences [36] from respiration [53] and non-neuronal effects [49,54].

### Correlation Computation

Temporal correlation computations for each participant per group utilized the time series of spontaneous BOLD fluctuations across 3 EPI runs (a 17.5 minute time series after concatenating the runs). Temporal correlations determined the probability that two seed regions were active at the same time, (e.g., the time series in the voxels in each seed region correlated with one another). We excluded the first 15 volume acquisitions from each run to assure magnetization equalization and to avoid artifacts associated with the start of scanner noises.

A first stage exploratory analysis considered temporal correlations between paired spherical seed regions centered on coordinates from parts of the brain that tinnitus might alter (Table 2). These included regions in auditory cortex and in visual and somatosensory cortex to determine whether changes in auditory cortex affected other sensory systems. We additionally selected seed regions in the attention network [33,34], because behavioral evidence indicated tinnitus disrupts attention [15,22,24]. These regions included components of the parieto-frontal dorsal network for selective, voluntary attention (bilateral posterior intraparietal sulcus, bilateral frontal eye fields, and right ventral intraparietal sulcus). Two selected ventral attention regions show activation when focus involuntarily shifts to salient, but unexpected events (right temporoparietal junction and right superior temporal sulcus). Four chosen fronto-insula cortex regions (right middle frontal gyrus, right anterior insula, and bilateral inferior frontal gyrus) included those involved in switching between the dorsal and ventral attention networks [33,34] or generally between control networks [55,56].

**Table 2:** Talairach atlas coordinates for selected seed regions

NETWORK	SEED REGION NAME	TALAIRACH COORDINATES		
		**X**	**Y**	**Z**

AUDITORY	Right Primary Auditory (RAud)	51	-21	9
	Left Primary Auditory Cortex (LAud)	-41	-26	7
VISION	Right Primary Visual (RV1)	11	-81	5
	Left Cuneus (LV2d)	-4	-85	19
SOMATOSENSORY	Right Postcentral Gyrus (RS1)	51	-18	44
	Left Parietal Operculum (LS2)	-35	-27	17
DORSAL ATTENTION	Left Posterior Intraparietal Sulcus (LpIPS)	-23	-66	46
(DAN)	Right Posterior Intraparietal Sulcus (RpIPS)	26	-58	52
	Left Frontal Eye Fields (LFEF)	-25	-8	50
	Right Frontal Eye Fields (RFEF)	27	-8	50
	Right Ventral Intraparietal Sulcus (RvIPS)	30	-80	16
VENTRAL ATTENTION	Right Temporoparietal Junction (rTPJ)	49	-50	28
(VAN)	Right Superior Temporal Sulcus (RSTS)	55	-50	11
ATTENTION CONTROL	Right Middle Frontal Gyrus (rMFG)	39	12	34
	Right Anterior Insula (RAI)	36	3	6
	Left Inferior Frontal Gyrus (LIFG)	-41	6	9
	Right Inferior Frontal Gyrus (RIFG)	45	-3	12

The time series from each seed region was the average across all voxels within the ~1 cm^3 ^spheres. A Fischer's computation converted correlation coefficients to Z-transforms [57]. Post hoc t-tests evaluated group differences in the strength of temporal correlations between paired seed regions using the Z-transforms from each group of participants.

### Group Contrast Analyses of Functional Connectivity Maps

In a second stage analysis, we computed functional connectivity maps for each of those seed regions whose paired temporal correlations had group differences with probabilities < .05. In the functional connectivity maps computed in each participant, computed correlations were between the time series averaged across all voxels in a selected seed region and the time series in each 2 mm^3 ^volume (voxel) in the brain [36,49,50,58].

All evaluations of the functional connectivity map for a particular seed region utilized voxel level Fisher's Z-transforms in each participant registered to a standard, population-average, cortical surface-based atlas (PALS-B12) [59]. The registration process involved creating participant-specific surfaces using FreeSurfer. Participant surfaces were then deformed to the distribution of nodes in the PALS-B12 atlas using an automated procedure to align nine anatomical landmarks [60], individually identified in a participant hemisphere and registered to the same landmarks in the average PALS-B12 atlas sphere. The deformation matrices obtained from landmark-based alignments created for each participant guided registration of Fisher Z-transform-scores from the nodes of each participant-specific surface to the PALS-B12 atlas nodes. Data registration between volume and surface space involved aligning the atlas coordinates of voxels to corresponding nodes with nearest neighbor coordinates in the PALS-B12 atlas [61]. Separately for each group, determination of the connectivity map for a seed region utilized computed means of surface registered Fisher transform Z-scores. A random effects Student's t-test [62] evaluated the null hypothesis of no significant distribution of Z-transforms across the group connectivity map. Displayed maps utilized two-tailed t-test results with probabilities of 0.05-0.005 (t = ± 2.1 and 3.3, 16df).

Next, we computed a *t *statistic at each surface node to assess the null hypothesis that the Fisher transform Z-scores for a seed region were comparable between the control and tinnitus groups. The *t *statistic was computed as the mean difference (control group Z-transform score minus tinnitus group Z-transform score) divided by the SEM difference. Results were visualized using probability thresholds of 0.05-0.002 (t = ± 2.1-3.3 for 32df). Positive t-test results occurred at nodes where control group connectivity had greater positive correlations and/or the tinnitus group had greater negative correlations. Negative t-test results occurred where control group connectivity had larger negative correlations or the tinnitus group had larger positive correlations.

We assessed the significance of clusters observed in the group contrast *t*-statistic maps with a threshold-free cluster enhancement (TFCE) method. The analysis evaluated whether a cluster in the *t *statistic map was large enough to be statistically significant without needing to specify a cluster size threshold semi-arbitrarily. The original implementation of the TFCE method was for volumetric data [63] and was recently adapted to data registered to surface-nodes [60].

For the current analysis, we first computed 5000 t-maps with each generated after randomly combining all participants and then equally dividing them into groups of 17. After minimal spatial smoothing of the t-maps across neighboring nodes, a transformation of the *t *statistic at each surface node produced a TFCE map for each t-map. Documentation is available at the following website: http:///brainvis.wustl.edu/wiki/index.php/Caret:Documentation:Statistics:TFCE_Implementation. The transformation included information about t map signal intensity, h, and extent (the number of contiguous nodes (p) with h at or above threshold). In the computation of a TFCE score at a node, the weight given to signal intensity and extent value was fixed using H = 2.0 and E = 1.0. The maximum TFCE score from each randomized t-map contributed to a distribution of TFCE scores, each representing a cluster of contiguous nodes. Clusters in the original *t *statistic map were judged significant at p = 0.05 where they corresponded to the 95^th ^percentile of the TFCE maximum score distribution.

### Evaluation of Data Quality

**Figure 1:**
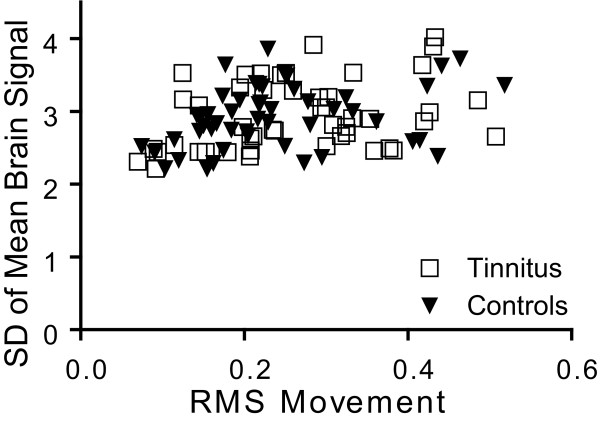
**Comparison between tinnitus and control participants for mean brain signals and head movements**. Scatter plot of standard deviation (STDev) of mean whole-brain signals vs. root-mean square (RMS) of head movements.

Spurious factors potentially affecting group functional connectivity results include excessive head movements and spurious magnetic signals. However, Figure 1 shows the tinnitus and control groups had comparable head movements (root mean square, RMS) and whole-brain variance in MR signal magnitudes. Two-tailed t-tests found no significant group differences in RMS or standard deviation of average brain magnetic signal amplitude (SD) (RMS: p = 0.23, SD: p = 0.84 df = 96).

## Results

### Temporal Correlation Matrix

Table 3 presents t-test probabilities for the differences between tinnitus and control groups in correlation magnitudes. The results were from 136 pairings of 17 different seed regions. Notably, tinnitus and controls had similar temporal correlations for nearly all seed pairings made with components of the dorsal attention network. One exception was for RvIPS vs. RTPJ; the tinnitus group had a greater negative correlation of -0.1 compared to a -0.01 correlation in controls. Only 10 seed region pairings (Table 3, underlined cells) had p < .05 suggesting possible group differences. We assessed functional connectivity differences between groups only for the 12 different seed regions making up these temporal correlation pairs. This second stage analysis included family-wide error corrections [63]. Significant clusters based on group differences occurred in 7/12 of these seed regions: LAud, RAud, RSTS, RV1, RAI, LIFG, and RIFG. Significant group differences in the functional connectivity analysis were absent for the remaining 5/11 including seed regions from the attention network: RvIPS and RTPJ, somatosensory system: RS1 and LS2 and visual system: LV2d. In each of the latter, the observed clusters found in the group contrast between connectivity maps were too small to pass the stringent error correction requirements of the TFCE permutation analysis.

**Table 3:** Temporal correlation matrix^a^

		DAN					VAN	Control					Sensory					
		LpIPS	RpIPS	LFEF	RFEF	RvIPS	RTPJ	RMFG	RSTS	LIFG	RIFG	RAI	LAud	RAud	RV1	LV2d	RS1	LS2
DAN	LpIPS		0.34	0.99	0.97	0.48	0.69	0.31	0.33	0.64	0.77	0.68	0.62	0.62	0.81	0.66	0.82	0.15
	RpIPS			0.52	0.90	0.51	0.31	0.45	0.74	0.72	0.47	0.17	0.12	0.44	0.87	0.69	0.21	0.29
	LFEF				0.25	0.88	0.40	0.09	0.46	0.39	0.16	0.66	0.26	0.57	0.71	0.63	0.18	0.15
	RFEF					0.89	0.49	0.85	0.49	0.59	0.50	0.77	0.50	0.89	0.44	0.36	0.88	0.72
	RvIPS						0.04	0.44	0.59	0.71	0.97	0.26	1.00	0.51	0.23	0.03	0.82	0.57
VAN	RTPJ							0.07	0.83	0.05	0.46	0.06	0.51	0.87	0.28	0.28	0.83	0.95
Control	RMFG								0.81	0.17	0.28	0.09	0.70	0.44	0.10	0.09	0.01	0.09
	RSTS									0.07	0.01	0.29	0.43	0.40	0.73	0.42	0.44	0.84
	LIFG										0.02	0.02	0.06	0.08	0.12	0.27	0.89	0.07
	RIFG											0.20	0.91	0.77	0.86	0.05	0.27	0.10
	RAI												0.02	0.09	0.59	0.03	0.87	0.03
Sensory	LAud													0.40	0.54	0.51	0.70	0.55
	RAud														0.02	0.31	0.92	0.70
	RV1															0.21	0.60	0.28
	LV2d																0.64	0.87
	RS1																	0.77
	LS2																	

^a^See Table 2 for identification of abbreviations

### Functional Connectivity for Auditory Network Seeds

**Figure 2:**
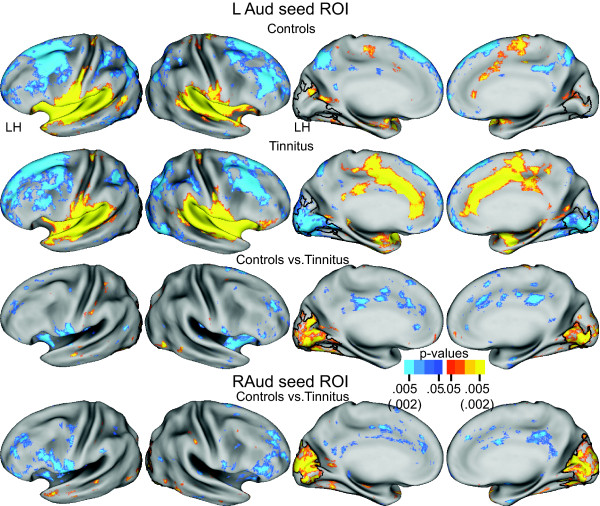
**Functional connectivity maps for a left primary auditory area (LAud) seed region centered in Heschl's gyrus**. Rows 1 and 2 show, respectively, random effect functional connectivity t-maps [62] for controls and tinnitus displayed on inflated views of the PALS-B12 atlas surface [59]. The distribution of positive and negative correlations between time courses in the seed vs. other brain locations painted, respectively, in yellow-orange and blue (scale: p value 0.05-0.005). Row 3 shows map of a t-test assessment per node of group differences in Fisher Z-transforms of correlations. Significant t-test results marked in yellow-orange for positive and blue for negative (scale: p value 0.05-0.002). Row 4 shows t-test results for contrast between functional connectivity maps for a right primary auditory area (RAud) seed region. Black borders surround significant cluster identified with the threshold free cluster extension analysis [63] after 5000 permutations of the t-test analysis and an alpha threshold < .05.

Functional connectivity based on auditory seed regions revealed differences between the groups in the network between auditory and occipital/visual cortex. In the tinnitus group, functional connectivity for a left primary auditory cortex seed (LAud) involved significant negative correlations throughout the medial aspect of bilateral occipital cortex (Figure 2, row 2, columns 3, 4). In the control group, the functional connectivity map for the LAud seed contained a few patches of positive correlations in medial occipital cortex (Figure 2, row 1, columns 3, 4). The TFCE permutation analysis of the t-test group contrast between controls compared to tinnitus showed a significant cluster whose borders extended from the occipital pole to the parietal occipital sulcus, with a slightly greater extent in the left hemisphere (Figure 2, row 3, columns 3, 4). The cluster centered across the calcarine sulcus, covering upper and lower banks and the adjoining cuneus and lingual gyri. The values were positive because of subtracting the negative correlations in tinnitus in the t-test, leading to positive values that added to the few positive correlations in controls. Group functional connectivity differences were similar for a right primary auditory cortex seed with the TFCE analysis of the group contrast similarly revealing a significant bilateral cluster covering the same portion of medial occipital cortex except for being slightly more extensive in the right hemisphere (Figure 2, row 4).

The functional connectivity maps also showed significant positive correlations between primary auditory cortex and the superior temporal plane, insula, inferior frontal gyrus, and cingulate cortex (Figure 2, rows 1, 2, columns 1, 2). The magnitudes and extent of these positive correlations was greater in tinnitus compared to controls resulting in negative value clusters in the t-test group contrast in the inferior frontal gyrus and rostral insula (Figure 2, rows 3, 4, columns 1, 2). However, these clusters were not large enough to pass the family-wide error corrections in the TFCE analysis.

Both groups had functional connectivity based on negative correlations between auditory seed regions and components of the default mode system (posterior cingulate, superior frontal, medial prefrontal, and lateral inferior parietal) and dorsolateral prefrontal. None of the clusters was significant in the TFCE analysis.

In summary, auditory cortex in participants with tinnitus had significant functional connectivity with occipital/visual cortex in which the correlations were negative. Controls mainly had connectivity based on positive correlations.

### Functional Connectivity for a Primary Visual Cortex Seed Region

**Figure 3:**
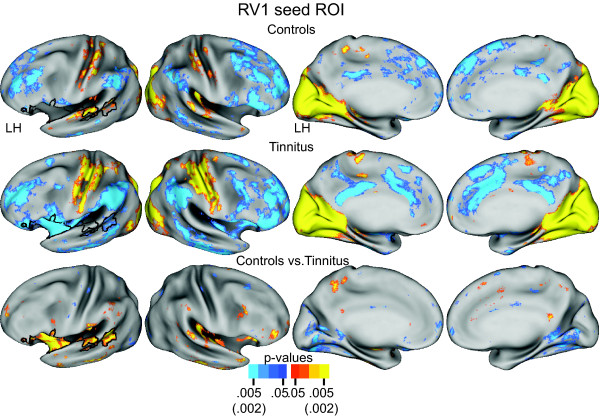
**Functional connectivity maps for a seed region in right primary visual area (RV1) centered within the calcarine sulcus**. Rows 1 and 2 show, respectively, random effect functional connectivity t-maps [62] for controls and tinnitus displayed on inflated views of the PALS-B12 atlas surface [59]. The distribution of positive and negative correlations between time courses in the seed vs. other brain locations painted, respectively, in yellow-orange and blue (scale: p value 0.05-0.005). Row 3 shows map of a t-test assessment per node of group differences in Fisher Z-transforms of correlations. Significant t-test results marked in yellow-orange for positive and blue for negative (scale: p value 0.05-0.002). Black borders surround significant cluster identified with the threshold free cluster extension analysis [63] after 5000 permutations of the t-test analysis and an alpha threshold < .05.

Functional connectivity for a right primary visual area seed (RV1) revealed differences between the groups that reciprocated the connectivity distinctions noted for seeds in auditory cortex. The RV1 seed was located in calcarine sulcal cortex, within the region of the significant clusters discovered with the auditory cortex seed regions. In the control group, functional connections included positive correlations in auditory cortex and a less extensive distribution of negative correlations in the temporoparietal junction, inferior frontal gyrus, and components of the default system (Figure 3, row 1). In the tinnitus group, functional connectivity for the RV1 seed involved significant negative correlations bilaterally in auditory cortex, temporoparietal junction, inferior frontal gyrus, and components of the default system (Figure 3, row 2). Both groups also showed significant positive correlations throughout occipital/parietal-occipital cortex and sparser connectivity with pericentral gyral cortex. The TFCE permutation analysis of the t-test group contrast between controls compared to tinnitus showed several significant clusters with borders in left hemisphere superior temporal gyral and sulcal auditory cortex, rostral insula, and adjoining inferior frontal gyrus (Figure 3, row 3, columns 1, 3, and 4).

In summary, negative correlations with auditory cortex characterized the connectivity associated with visual cortex seed regions in participants with tinnitus. These functional connectivity differences again indicated a phase reversal in the resting state activity between the visual and auditory systems in tinnitus. Additionally, this phase reversal extended to parts of the attention and default mode networks.

### Functional Connectivity for Seed Regions Involved in Executive Control of Attention

#### Right Anterior Insula

**Figure 4:**
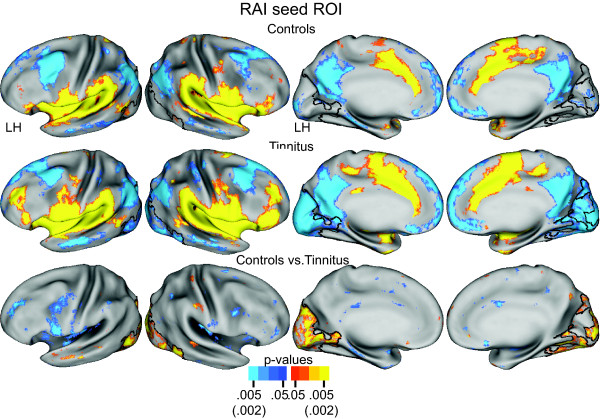
**Functional connectivity maps for a seed region in right anterior insula (RAI)**. Rows 1 and 2 show, respectively, random effect functional connectivity t-maps [62] for controls and tinnitus displayed on inflated views of the PALS-B12 atlas surface [59]. The distribution of positive and negative correlations between time courses in the seed vs. other brain locations painted, respectively, in yellow-orange and blue (scale: p value 0.05-0.005). Row 3 shows map of a t-test assessment per node of group differences in Fisher Z-transforms of correlations. Significant t-test results marked in yellow-orange for positive and blue for negative (scale: p value 0.05-0.002). Black borders surround significant cluster identified with the threshold free cluster extension analysis [63] after 5000 permutations of the t-test analysis and an alpha threshold < .05.

The network for the right anterior insula (RAI) seed in both groups involved significant positive correlations throughout adjoining parts of the auditory cortex along the superior temporal plane (Figure 4, row 1, 2, columns 1, 2). The RAI functional connectivity maps included group distinctions in occipital cortex. In the control group, functional connectivity in occipital cortex was scarce (Figure 4, row 1). In the tinnitus group, functional connectivity involved significant negative correlations bilaterally in medial and lateral occipital cortex (Figure 4, row 2). The TFCE permutation analysis of the t-test group contrast showed a significant cluster whose borders extended medially and laterally over the whole of occipital cortex (Figure 4). Additionally, functional connectivity with positive correlations in the left inferior frontal gyrus was greater in the tinnitus group, leading to a cluster with a negative t-test value (Figure 4, row 3, column 1). This cluster, however, did not pass the TCFE significance threshold.

#### Left Inferior Frontal Gyrus Seed

**Figure 5:**
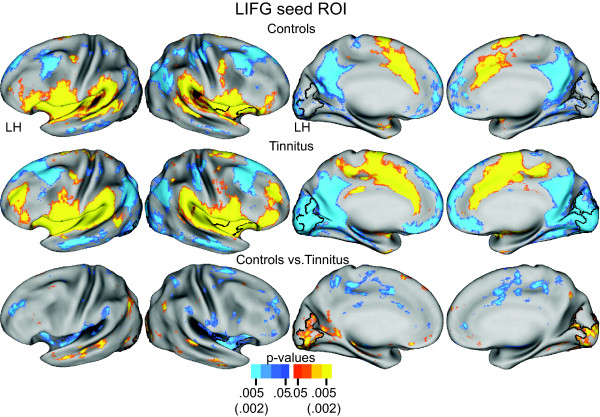
**Functional connectivity maps for a seed region in left inferior frontal gyrus (LIFG)**. Rows 1 and 2 show, respectively, random effect functional connectivity t-maps [62] for controls and tinnitus displayed on inflated views of the PALS-B12 atlas surface [59]. The distribution of positive and negative correlations between time courses in the seed vs. other brain locations painted, respectively, in yellow-orange and blue (scale: p value 0.05-0.005). Row 3 shows map of a t-test assessment per node of group differences in Fisher Z-transforms of correlations. Significant t-test results marked in yellow-orange for positive and blue for negative (scale: p value 0.05-0.002). Black borders surround significant cluster identified with the threshold free cluster extension analysis [63] after 5000 permutations of the t-test analysis and an alpha threshold < .05.

The functional connectivity maps for the left inferior frontal gyrus seed (LIFG) partially resembled those for RAI. Thus, both groups had significant positive correlations in auditory cortex along the superior temporal plane (Figure 5, row 1, 2, columns 1, 2). Furthermore, the LIFG network included group connectivity distinctions in occipital cortex. In the control group, functional connections in occipital cortex were scarce (Figure 5, row 1). In the tinnitus group, functional connectivity involved significant negative correlations bilaterally in medial occipital cortex (Figure 5, row 2). The TFCE permutation analysis of the t-test group contrast showed a significant cluster with borders located in medial occipital cortex (Figure 5). Both groups also showed significant positive correlations in the rostral insula cortex. These had greater correlation magnitudes and spatial extents in the tinnitus group. The TFCE analysis identified this difference as a significant cluster in the right anterior insula cortex (Figure 5, row 3, column 2), thus, reciprocating the connectivity in the left inferior frontal gyrus observed for the RAI seed (Figure 4).

In summary, seed regions in the right anterior insula and left inferior frontal gyrus of the tinnitus group had significantly greater functional connectivity that positively correlated with resting state activity in auditory cortex and negatively correlated with parts of occipital cortex.

## Discussion

The participants with tinnitus also were in a study that evaluated staged periods of treatment with rTMS [46]. However, the current functional connectivity analysis focused exclusively on pre-treatment, baseline activity.

### Chronic Tinnitus, a Possible Unique Usage Factor

A hypothesized mechanism for changes in functional connectivity is that altered usage induces changes in synaptic efficacy [14] that possibly contribute to neural plasticity in tinnitus [5]. If the underlying cause of tinnitus was hearing loss that then leads to persistent bothersome phantom sounds, the latter might be a usage factor consistent with connectivity differences in tinnitus. The usage factor might be adaptations to living with persistent endogenous noise that involve attempts to damp-down or redirect attention away from the salience of these sounds. This would be a usage factor that could lead to re-organization of affected synapses connected through structural or indirect linkages between network components [42]. Such re-organized synaptic efficacy whose purpose is to ward-off the distractions from phantom sounds possibly underlies the observed connectivity differences in participants with tinnitus.

A consideration from the observed different connectivity patterns was that they arose as a consequence of living with tinnitus rather than as its cause. Thus, the functional connectivity differences in tinnitus possibly were in the affected regions rather than the instigators of phantom noises. Hypothetically, adaptive compensations to tinnitus altered functional connectivity. Alternatively (or additionally), the observed functional connectivity differences in tinnitus were maladaptive manifestations that perpetuated some associated neuropsychiatric sequelae, if not the ringing itself.

Peripheral auditory deafferentation effects in tinnitus patients [4,6-9] might be an alternative explanation for the observed connectivity changes as opposed to cognitive induced usage factors. Some peripheral damage was probable in the studied tinnitus group because they had moderate to severe hearing loss for the 8 kHz tones. Such a loss possibly reflected profound peripheral deafferentation that could have resulted in altered maps in auditory cortex [64]. Total deafferentation in individuals with complete blindness show functional connectivity changes [65] and that also lead to cross-modal activation of visual cortex by non-visual inputs [66]. These changes, however, occur where blindness is congenital or present before age 3. Cross-modal changes are less prevalent in adults with adventitious, late-onset blindness [66]. Tinnitus is an adult onset disease and the studied population still had relatively normal audiograms for lower frequencies. Although deafferentation induced plasticity occurs in adults, the changes frequently result in expanded cortical representation for the surviving modal specific inputs [10,11] even in tinnitus patients [64]. Consequently, the suggestion that the observed functional connectivity differences in tinnitus directly arose from deafferentation induced changes for high frequency sounds seems like a less parsimonious explanation. This speculation particularly does not reflect that sensory loss in adults leads to more limited, local changes in modal specific cortex whereas the observed functional connectivity alterations involved widespread consequences in several cortical regions. However, partial hearing loss might be contributory to changes in synaptic efficacy that then more prominently change from cognitive factors.

The network changes in the auditory, visual, attention, and control cortices observed with tinnitus reflected behavioral disruptions previously noted in patients with a bothersome tinnitus history. Patients with other chronic neuropathology also have altered functional connectivity [37-40]. In each instance, connectivity distinctions were unique. Thus, the current findings in tinnitus were also probably unique and distinguishable from connectivity distinctions in other chronic clinical conditions, like pain or traumatic stress disorders.

### Connectivity Differences based on Positive and Negative Correlations

Major group differences in functional connectivity involved the negative correlations in the linkages between auditory and visual networks. Negative correlations indicate that when resting-state spontaneous BOLD response magnitudes increased in one location they decreased in the linked location. In tinnitus, activity in a primary auditory cortex seed region negatively correlated with activity in occipital/visual regions. Reciprocally in the tinnitus group, connectivity for the right primary visual cortex negatively correlated with activity in auditory cortex. These results imply that increases in activity in auditory and visual networks reciprocally caused a decrease of BOLD response magnitudes in each other.

Prior examples of blood flow decreases in sensory systems occurred in the cortex that normally processed the modality that was irrelevant to the engaged task [18,67,68]. These negative responses might reflect activation of inhibitory circuits through functional connectivity from the task-relevant modality to the cortical representation of the non-relevant sensory system [18]. In tinnitus, the phantom sounds might act to decrease activity in visual cortex because the visual system is "irrelevant" to processing the apparition of sounds in tinnitus. However, the distribution of negative correlations associated with the auditory or visual cortex seed regions included non-sensory cortex. Consequently, the observed differences between groups might reflect extensive changes in network activity additional to neural processing in an appropriate sensory cortex.

Prior functional connectivity studies showed system-wide differences in the distribution of negative/positive correlations when analyzing resting-state activity with a global brain signal regressed out of the computations [41]. Attention and default mode networks showed divergent fluctuations in spontaneous resting state activities characterized by negative temporal correlations [49]. These phase reversed BOLD response magnitudes between attention and default mode networks were labeled "anti-correlated" [41,49], suggesting that activity dedicated to events in the outside world (attention) necessarily differed from endogenous autobiographical references (DMN). In tinnitus, reciprocal connectivity based on negative correlations between activities in visual and auditory sensory networks possibly reflected comparable changes in brain functions leading to dissociation between the auditory and visual systems. Thus, a person with tinnitus might need to dissociate or suppress involuntary attention to the auditory system when processing visual inputs [26-28].

Observations of blood flow decreases or negative BOLD signals during tasks initially aided discovery of a default mode network (DMN) [20,69,70]. DMN is active at rest [16,19], particularly during self-referential behavior [71], but shows decreased activity during any task. In the current study, DMN was comparable between groups, indicating preservation of normal autobiographical reveries, recollections, and planning in tinnitus patients despite the presence of persistent phantom auditory sensations.

### Phantom Noises as a Salience or Conflict Feature

Connectivity differences between groups also included cortex regions important for switching between conditions that conflict or have different salience [34,55,56]. Thus, the differences in tinnitus connectivity between the RV1 seed region and fronto-insular and attention network components might reflect an adaptation to reduce the salience of phantom noises in tinnitus and maintain attention on non-auditory events. Endogenous ringing sounds involuntarily capture attention in those with bothersome tinnitus. The hypothesized consequence of these effects is depletion in cognitive resources [15]. These sounds are not cued and do not require any specific goal-directed response. The exception is the goal of not allowing tinnitus to interfere with normal stimulus and cognitive processing. However, there is an on-going conflict between the tinnitus and other processing that partially relates to the salience of the two conditions. These factors of conflict and salience might underlie the connectivity differences noted in the inferior frontal gyrus and rostral insular cortex [55,56].

The functional connectivity in inferior frontal and right anterior insula cortex might potentially regulate cognitive switching in conflict situations [56] or influence switching on the basis of salience for conditions that capture attention selectively compared to involuntarily [33,34,55]. Connectivity with fronto-insular cortex involved negative and positive correlations, respectively, from visual and auditory cortex. In one hypothetical model, the fronto-insular cortex is part of a salience network that drives switching by a central executive control network important to maintaining and adjusting attention [55]. In reference to tinnitus, the issue concerns the salience of phantom noise in contrast with some task-based condition. In this context, fronto-insular cortex might initiate resolution of conflicts between the salience of phantom noises and the more important non-tinnitus conditions that involve task specific, possibly visual processing. Consistent with the latter notion is evidence of activation of inferior frontal gyral and anterior insular regions during visual Stroop tasks with conflicts between congruent vs. incongruent or neutral conditions [56]. Phantom tinnitus noises might then represent the incongruent condition that conflict with processing visual inputs that represent congruent conditions. However, how fronto-insular cortex acts through phase-reversed resting-state visual cortex activity in tinnitus is unknown. In participants with tinnitus, negative correlations dominated the functional connectivity with the visual system for RAI and IFG. RAI and IFG had stronger positively correlated connectivity with primary auditory cortex, suggestive of a reinforced suppression of visual processes in tinnitus, which is an opposite effect from resolving conflicts that arise from tinnitus.

In another model, the rostral insula, inferior frontal, and posterior middle frontal cortex on the right act as executive control components in the attention system that regulate dorsal and ventral attention networks, which lack direct interconnections [33,34,72]. Activity in these control components might affect connectivity with the dorsal and ventral attention networks in the tinnitus group. However, the current results only showed connectivity distinctions between RV1 and rTPJ.

The right TPJ is a component of the salience, stimulus-driven ventral attention network [33,34]. Prior studies reported activation of rTPJ when attention reoriented to unexpected yet behaviorally noticeable stimulation [73-75]. However, suppresion of rTPJ happened during stimulation that was not relevant to the goals of a task [76,77] or was a cued reorientation of attention [72]. In the current study, rTPJ showed a greater spatial extent of negative correlations in the tinnitus compared to the control group with the RV1 seed. Tinnitus is definitely irrelevant to all behavioral and cognitive searches. Goal-directed cognitive effort to examine some visual input and ignore tinnitus might thus necessitate spatially more extensive phase-reversed activity in rTPJ to prevent reorienting of attention to the tinnitus percept. This hypothesis was supported by previous work showing that goal-directed behavior, such as participating in visual search [76,77], suppressed rTPJ activity. Functional connectivity through negative correlations between RV1 and rTPJ might instantiate deactivation in tinnitus to prevent reorientation to distracting endogenous phantom sounds, which are not behaviorally relevant.

Connectivity for components of the dorsal attention system was similar in tinnitus and control groups, indicating that the presence of tinnitus did not affect the ability to voluntarily focus attention, a factor critical to behavioral treatment strategies for tinnitus [2,24,25].

### Technical Factors

Two reviews of the neurobiology of tinnitus expressed concerns that divergent results amongst prior neuroimaging studies possibly reflected varied demography of tinnitus patients including differences in age and/or hearing loss, case studies or sample sizes of < 10 and only fixed as opposed to random effect statistics, non-equivalent data that also lacked spatial resolution, lack of data from control groups, and theoretical comparisons between microscopic measurements in animals and macroscopic imaging data from humans [3,7]. Many of these issues were not present in the current study. The sample was 34, equally divided into participants with and without bothersome tinnitus. Although the tinnitus group contained more males than the control group, this difference was not great. Furthermore, gender differences are not known to influence tinnitus symptoms or be a factor in the cortical regions found to show connectivity differences, although gender differences possibly can influence processing noise stimuli in primary auditory cortex [7]. Age, which influences hearing thresholds, closely matched between the two groups. The pure tone average threshold for both groups was ~25 dB HL for frequencies between 1 and 3 kHz. The tinnitus group, however, had hearing deficits for tones > 8 kHz, but hearing loss is a known precipitating factor leading to tinnitus, and having tinnitus was an inclusion factor in the current study. Because hearing loss in the studied tinnitus group was not extreme, we suggest that the most parsimonious basis for connectivity differences was the experienced persistent phantom noises as opposed to hearing deficit differences between the groups. Participants with tinnitus were similar in having bothersome symptoms with moderate to severe THI scores and loudness that varied from 5 to 9, all lacked hyperacusis, and they were free of severe depression.

Within and between group comparisons benefited from optimized spatial resolution of the imaging data. Surface rendering of the cortical surfaces respected the fiduciary anatomy of each hemisphere. Additionally, identification of standard anatomical landmarks in each hemisphere enabled spatial registration of data within and across groups with < 3% distortion to a common atlas space wherein statistical comparisons were executed [59]. Our analyses also included assessment of each case followed by random effect statistics of group data and family-wide error corrected statistics of group contrasts. The stringency of the TFCE permutation analysis might have been prone to Type II errors [63], suggested by evidence of clusters in the default mode system and temporoparietal junction that did not pass the cluster size significance threshold.

## Conclusions

Tinnitus patients showed altered functional connectivity for auditory and visual networks compared to age-matched controls. The connectivity differences between tinnitus and controls concerned negative correlations in tinnitus, indicating that when resting-state spontaneous BOLD response magnitudes increased in auditory cortex they decreased in visual cortex. Reciprocally in the tinnitus group, connectivity for the right primary visual cortex negatively correlated with activity in auditory cortex. These results imply that increases in activity in auditory and visual networks reciprocally decreased BOLD response magnitudes in each other. The functional connectivity in inferior frontal and right anterior insula cortex negatively correlated with the right primary visual cortex and positively correlated with auditory cortex. The fronto-insular cortex potentially provides executive control over switching attention between conflicting salient phantom noises and other conditions [33,34,55,56]. The differences in tinnitus connectivity might reflect an adaptation to reduce the salience of phantom noises in tinnitus and maintain attention on non-auditory events. Thus, goal-directed cognitive efforts to examine visual inputs and ignore tinnitus might necessitate phase-reversed activity to block reorienting of attention to the salient, but irrelevant tinnitus percept. These differences in tinnitus were consistent with the hypothesis that chronically accommodating to persistent bothersome phantom sounds induced changes in synaptic efficacy between functionally connected network components.

## Authors' contributions

HB wrote the paper, conceived of the study design and data analysis, and interpreted the findings. AW contributed to the acquisition, analysis, and interpretation of the data. MB contributed to the analysis and interpretation of the data. JN contributed to the conception of the study and acquisition of the data. KSG conceived of the study. JFP contributed to the conception and design of the study.

All authors read and approved the manuscript.
